# Moral Distress in Medical Directors Barriers to Facilitating Evidence Based Improvements in Practice

**DOI:** 10.1093/geroni/igaf122.009

**Published:** 2025-12-31

**Authors:** Catherine MacDonald, Annie Rhodes, Leland Waters

**Affiliations:** Virginia Commonwealth University, Richmond, Virginia, United States; Virginia Commonwealth University, Richmond, Virginia, United States; Virginia Commonwealth University, Richmond, Virginia, United States

## Abstract

In nursing homes, the Medical Director (MD) serves a leadership role, as such the voice of the MD cultivates the quality of resident care, and sets standards for workforce training. MDs are responsible for coordinating care, implementing and evaluating care policies, and ensuring adherence to clinical standards. MDs use their voice in clinical oversight, the influence of administrative decisions, quality assurance, and process improvement initiatives. MDs, like other nursing home staff, must navigate organizational and regulatory barriers that accompany quality resident care, and as such, MDs are at risk for compassion fatigue and moral distress arising from systemic barriers. These conflicting institutional priorities affect both residents and staff. MDs navigate these challenges personally while simultaneously being required to use their voice and authority to serve on the leadership team. This session explores the intersection of moral distress and the MD role in the nursing home. Semi-structured interviews with MDs are analyzed through dialogical narrative analysis, with an emphasis on voice, and close, annotative top-to-bottom readings of the transcripts. The exploratory, in-depth, and narrative nature of long-form interviews provides a compelling ­narrative method that can elicit rich and in-depth data and understanding and participant voice. While the MDs share their voices from different facilities, their joint narratives provide unified accounts of their industry. The experiences of the MDs about compassion fatigue, moral distress and the organizational barriers and regulatory barriers that they encounter will be used to provide insight on how to improve person-centered care in the nursing home.

